# Dialysis recovery time: associated factors and its association with quality of life of hemodialysis patients

**DOI:** 10.1186/s12882-022-02926-0

**Published:** 2022-09-01

**Authors:** Mohamed Mamdouh Elsayed, Montasser Mohamed Zeid, Osama Mohamed Refai Hamza, Noha Mohamed Elkholy

**Affiliations:** Nephrology and Internal Medicine Department, Faculty of Medicine, Alexandria University, Alkhartoom square, El azareeta, Alexandria, 21131 Egypt

**Keywords:** Dialysis recovery time, Malnutrition, Quality of life, Hemodialysis

## Abstract

**Introduction:**

Post-dialysis fatigue is a common and distressing complaint in patients on hemodialysis (HD). The dialysis recovery time (DRT) is a recent and reliable method of Post-dialysis fatigue assessment. We aimed to identify factors affecting the DRT and its relation with HD patients’ quality of life.

**Material and methods:**

This is a cross-sectional study carried out on end-stage renal disease patients on regular HD. All participants underwent detailed history taking and complete physical examination, and data on dialysis and laboratory investigations were also collected. Patients were asked “How long does it take you to recover from a dialysis session?” to calculate the DRT. We used the Malnutrition-Inflammation Score (MIS) and KDQOL-36 questionnaire to assess patients’ nutritional status and quality of life, respectively.

**Results:**

Two hundred and ten patients were screened and 191, with a median age of 47 years, completed the study. Patients had a median DRT of 300 minutes (range: 0.0–2880.0), with 55% of patients reporting a DRT of > 240 minutes and 22.5% of them reporting a DRT of < 30 minutes. Patients had a median MIS score of 7 (range: 0–17). There was a statistically significant negative relation between the DRT and symptom/ problem list (*p* < 0.001), effects of kidney disease (*p* < 0.001), burden of kidney disease (*p* < 0.001), SF-12 physical composite (*p* = 0.001), and SF-12 mental composite (*p* < 0.001) of KDQOL. The results of multivariate analyses showed that dialysate Na (*p* = 0.003), and the number of missed sessions (*p* < 0.001) were independently correlated with the DRT.

**Conclusions:**

Decreased dialysate Na, and increased number of missed sessions were predictors of prolonged DRT. Patients with prolonged DRT were associated with poorer quality of life. Further randomized clinical trials are needed to assess strategies to minimize the DRT and, perhaps, enhance clinical outcomes.

**Trials registration:**

ClinicalTrials.gov Identifier: NCT04727281. First registration date: 27/01/2021.

## Introduction

Fatigue is a well-known and frequent symptom in end-stage renal disease (ESRD) patients after hemodialysis (HD) sessions [1]. The prevalence of post-dialysis fatigue ranges from 60 to 97% [2]. Many factors, including malnutrition, anemia, inflammatory state, inadequate dialysis and the ultrafiltration rate (UFR), have been incriminated in the pathogenesis [3]. The assessment of fatigue can be challenging for physicians due to the lack of a clear case definition. However, early recognition is essential because several treatable causes can be easily identified [4].

Several methods of assessing post-dialysis fatigue have been proposed; however, till now, none has been defined in guidelines as an optimal method [5]. However, the dialysis recovery time (DRT) has recently been an easy, reliable, and validated method of assessment [6]. It entails asking patients “How long does it take you to recover from a dialysis session?” [7]. Davenport et al. found that the DRT was ≥1 hr. in more than 75% of patients, and was associated with depression, and post-dialysis hypotension [8]. Other researchers reported an association between the DRT and UFR, dialysis adequacy, comorbidities [9, 10]. Prolonged DRT restricts the ability of the patients to perform their daily activities [11]. The recovery period after HD is more important for many patients than being hospitalized [12]. Prolonged DRT is associated with post-dialysis fatigue [13], decreased QOL, and increased risk of mortality [14, 15].

The health-related QOL (HRQOL) is a vital outcome for HD patients [16]. Both subjective and objective measures are used to assess HRQOL in patients with chronic kidney disease (CKD) and ESRD [17]. The National Quality Forum selected the Kidney Disease Quality of Life Short Form survey (KDQOL™-36) as the tool of choice for assessing this outcome in adult ESRD patients [18]. Fatigue is considered an important cause of poor QOL in HD patients [19]. ESRD patients with reduced QOL are more predisposed to morbidity and mortality [20].

Due to the limited research and non-conclusive findings about the DRT, we aimed in this study to identify factors affecting the DRT and assess the association between DRT and QOL in patients on HD.

## Patients and methods

### Study

This is a cross-sectional study carried out on patients from the dialysis units in the Alexandria University Hospitals. We included ESRD patients who had been assigned to regular long-term HD (thrice-weekly, four-hour HD sessions for more than 90 days), aged ≥18 years, were able to read and write, and were in a perfect mental health. We excluded patients who were unable to fill out the questionnaires because of hearing or reading problems and those with dementia, actual instability of clinical conditions requiring hospitalization, liver failure, and active cancer. We also excluded those who experienced a decline in the level of consciousness during HD sessions. The trial was registered on Clinicaltrials.gov (NCT04727281).

### Methods & Study outcomes

All patients included in the study were subjected to detailed history taking with emphases on demographic data, the cause of ESRD, the presence of comorbid conditions, the vintage of HD and vascular access. Thorough physical examination with a special focus on pre- and post-HD blood pressure, body temperature, patient’s interdialytic weight, and body mass index (BMI). Dialysis-related data, including the modality of HD, type of dialyzer, blood flow rate (ml/min), ultrafiltration rate (UFR) (ml/hr), type of anticoagulation administered, dialysate temperature, dialysate Na, assessment of dialysis adequacy using single-pool Kt/V Daugirdas formula (second generation) [21], HD session duration, and the number of missed sessions per month, were collected.

### Dialysis recovery time

The patients’ responses to the single open-ended question, “How long does it take you to recover from a dialysis session?,” were converted to a number of minutes as follows [7]:

- Answers given in minutes were recorded directly.
- Answers in hours were multiplied by 60.
- Variants of “half a day,” including the “next day,” were given a value of 720 min.
- Variants of “one day” were given a value of 1440 min.
- Variants of “more than a day” were given a value of 2160 min (36 h).

### Assessment of nutritional status

The Malnutrition-Inflammation Score (MIS) was used to assess each patient’s nutritional status [22]. The MIS has four sections (nutritional history, physical examination, BMI, and laboratory values) and ten components [23]. Each component has four levels of severity, ranging from 0 (normal) to 3 (severely abnormal). The sum of all 10 MIS components can range from 0 (normal) to 30 (severely malnourished), with a higher score reflecting a more severe degree of malnutrition and inflammation. The five nutritional history-based components include weight change, dietary intake, gastrointestinal symptoms, functional capacity, and comorbid conditions. The two physical examination components consist of an assessment of subcutaneous body fat and signs of muscle wasting. In addition to the foregoing seven subjective global assessment (SGA)-based components, the three MIS-unique sections include BMI, serum albumin level, and the serum total iron binding capacity (TIBC), the four increments of which are also scored from 0 through 3. The assessment was done after the dialysis session by the study investigators who were trained on how to calculate the MIS by the hospital nutritional specialist.

### Quality of life assessment using the kidney disease quality of life 36 (KDQOL-36) short form

Each patient’s HRQOL was assessed using the validated Kidney Disease Quality of Life-36 (KDQOL-36): https://www.rand.org/health-care/surveys_tools/kdqol.html [24]. The KDQOL™ -36 is a short form that includes the SF-12 as generic core plus the burden of kidney disease, symptoms/problems of kidney disease, and effects of kidney disease (EKD) scales from the KDQOL-SF™v1.3. The KDQOL™-36 contains five subscales: the Physical Component Summary (PCS), Mental Component Summary (MCS), Burden of Kidney Disease (BKD), Symptoms and Problems of Kidney Disease (SPKD), and EKD. The first two subscales are a general measure of patients’ HRQOL, whereas the last three assess issues specific to patients with ESRD or earlier stages of CKD [18]. We used an Arabic-translated version of the KDQOL-SF1.3, which was previously found to be reliable and validated to assess the HRQOL of ESRD patients for the common questions in the used score and translated the remaining questions [25].

The standard scoring program of the KDQOL-36™ is based on a Microsoft Excel spreadsheet and includes information about the computation method: https://www.rand.org/health-care/surveys_tools/kdqol.html. Scores for each dimension range from 0 to 100, with higher scores reflecting better HRQOL [26].

### Laboratory investigations

Pre-dialysis blood samples and post-dialysis serum urea were obtained on a mid-week day with their scheduled HD session. Hemoglobin, serum sodium, serum potassium, serum creatinine, blood urea, serum phosphorus, serum calcium, serum PTH, serum albumin, CRP, and TIBC were measured.

### Statistical analysis

Data were entered and analyzed using IBM SPSS software package version 20.0. (Armonk, NY: IBM Corp) Qualitative data were presented using frequencies and percentages. The Kolmogorov-Smirnov test was used to verify the normality of quantitative data distribution. Quantitative data were presented using the range (minimum and maximum), mean, standard deviation, median, and interquartile range (IQR) depending on whether the data distribution was normal or skewed. The threshold for statistical significance was set at *P* = 0.05. The Mann-Whitney test was used to skewed quantitative data between the study groups. The Kruskal–Wallis test was used to compare skewed quantitative data between more than two study groups. Spearman’s coefficient was used to measure correlations between two skewed quantitative variables. Univariate and multivariate linear regression analysis was performed to examine the relationship between DRT and various factors.

## Results

### Baseline characteristics of patients

Two hundred and ten patients were screened. Fifteen of them did not meet our inclusion criteria and four refused to participate. A total of 191 patients were included in the study. Their median age was 47.0 years (18.0–80.0); 58% of them were males, and they had a median dialysis vintage of 5.0 years (0.25–31.0). Up to 86.4% of them underwent HD via an arterio-venous fistula (AV fistula). Also, 55.5% were hypertensive, 13.1% were diabetics, and 19.4% had hepatitis C. The clinical characteristics and dialysis-related data of the patients are presented in Table 1.

**Table 1:** Clinical characteristics and dialysis-related data of patients (*n* = 191)

Parameter	Overall
	No (%), Mean ± SD or Median (Min.-Max.)
Age (years)	47.0 (18.0–80.0)
Sex	
Male	112 (58.6)
Female	79 (41.4)
Vintage of HD (years)	5.0 (0.25–31.0)
Smokers	36 (18.8)
MAP (mm/Hg)	
- Pre session	98.30 (50.0–153.0)
- Post session	93.0 (37.0–163.0)
Main comorbidities	
- Hypertension	106 (55.5)
- HCV	37 (19.4)
- DM	25 (13.1)
- IHD	21 (11)
- COPD	12 (6.3)
- HF	8 (4.2)
Dialysis modality	
HD	181 (94.8)
HDF	10 (5.2)
HD schedule	
Morning	98 (51.3)
Afternoon	78 (40.8)
Evening	15 (7.9)
Vascular access	
-AV fistula	165 (86.4)
-Cuffed catheter	24 (12.6)
-Graft	2 (10)
HD prescription	
High flux dialyzer	190 (99.5)
Dialyzer surface area (m^2^)	1.80 (1.70–2.20)
Blood flow rate (ml/min)	300.0 (220.0–450.0)
Session duration (hr.)	4.0 (3.0–5.0)
UF volume (ml/session)	3000.0 (0.0–6500.0)
UF rate (ml/hr.)	750.0 (0.0–1625.0)
Dialysate temperature (°c)	36.50 (35.0–37.20)
Dialysate Na (mmol)	140.0 (130.0–143.0)
Dialysate flow (ml/hr)	650.0 (400.0–800.0)
KT/V Daugridas	1.32 (0.42–2.14)
K (mEq/L)	5.40 (2.30–7.80)
Na (mEq/L)	133.0 (121.0–141.0)
TIBC (mcg/dl)	230.0 (91.0–481.0)
Hemoglobin (g/dl)	9.83 ± 1.57
Serum albumin (g/dl)	3.90 (2.40–5.0)
Serum calcium (mg/dl)	9.0 (6.0–12.10)
Serum phosphorus (mg/dl)	5.64 ± 1.46
Serum PTH (pg/ml)	355.0 (1.20–3850.0)
CRP (mg/l)	9.0 (1.0–208.70)

Normally quantitative data was expressed as Mean ± standard deviation (SD) while not normally quantitative data was expressed as Median (Min.–Max.), or absolute numbers as appropriate

*AV fistula* arterio-venous fistula, *CRP* C reactive protein, *COPD* chronic obstructive lung disease, *HCV* hepatitis C virus, *DM* diabetes mellitus, *HD* hemodialysis, *HDF* hemodiafiltration, *Kt/V* measuring dialysis adequacy, *HF* heart failure, *IHD* ischemic heart disease, *MAP* mean arterial pressure, *PTH* parathyroid hormone, *SGPT* serum glutamic pyruvic transaminase, *TIBC* total iron binding capacity, *UF* ultrafiltration

### DRT, nutritional assessment, and HRQOL assessment

The median DRT in our patients was 300 minutes (range: 0.0–2880.0), with 55% of patients reporting a DRT of > 240 minutes and 22.5% of them reporting a DRT of < 30 minutes. Regarding the nutritional status, patients had a mean BMI of 26.17 ± 5.36 kg/m^2^ and a median MIS score of 7 (range: 0–17). Regarding the KDQOL™-36 subscales, the “symptom problem” list median score was 66.67 (6.25–100.0), the “EKD” median score was 67.86 (0.0–100.0), and the “Burden of kidney disease” median score was 37.50 (0.0–100.0). The studied cases scored a median value for the SF-12 “physical composite” and the SF-12 “mental composite” of 35.35 (28.94–42.24) and 45.45 (19.06–65.0), respectively (Table 2).

**Table 2:** DRT, nutritional assessment, and HRQOL assessment (*n* = 191)

Parameter	Overall No (%), Mean ± SD or Median (Min.-Max.)
DRT (min)	300.0 (0.0–2880.0)
0–30	43 (22.5)
> 30–60	2 (1)
> 60–120	13 (6.8)
> 120–240	28 (14.7)
> 240	105 (55)
Nutritional parameters	
- BMI (kg/m^2^)	26.17 ± 5.36
- MIS	7.0 (0.0–17.0)
KDQOL subscales	
- Symptom/ problem list	66.67 (6.25–100.0)
- Effects of kidney disease	67.86 (0.0–100.0)
- Burden of kidney disease	37.50 (0.0–100.0)
- SF-12 physical composite	35.35 (28.94–42.24)
- SF-12 mental composite	45.45 (19.06–65.0)

Normally quantitative data was expressed as Mean ± standard deviation (SD) while not normally quantitative data was expressed as Median (Min.–Max.), or absolute numbers as appropriate

*BMI* body mass index, *DRT* dialysis recovery time, *MIS* Malnutrition-inflammation score, *KDQOL* Kidney disease quality of life

### Factors affecting the DRT

**Fig. 1:**
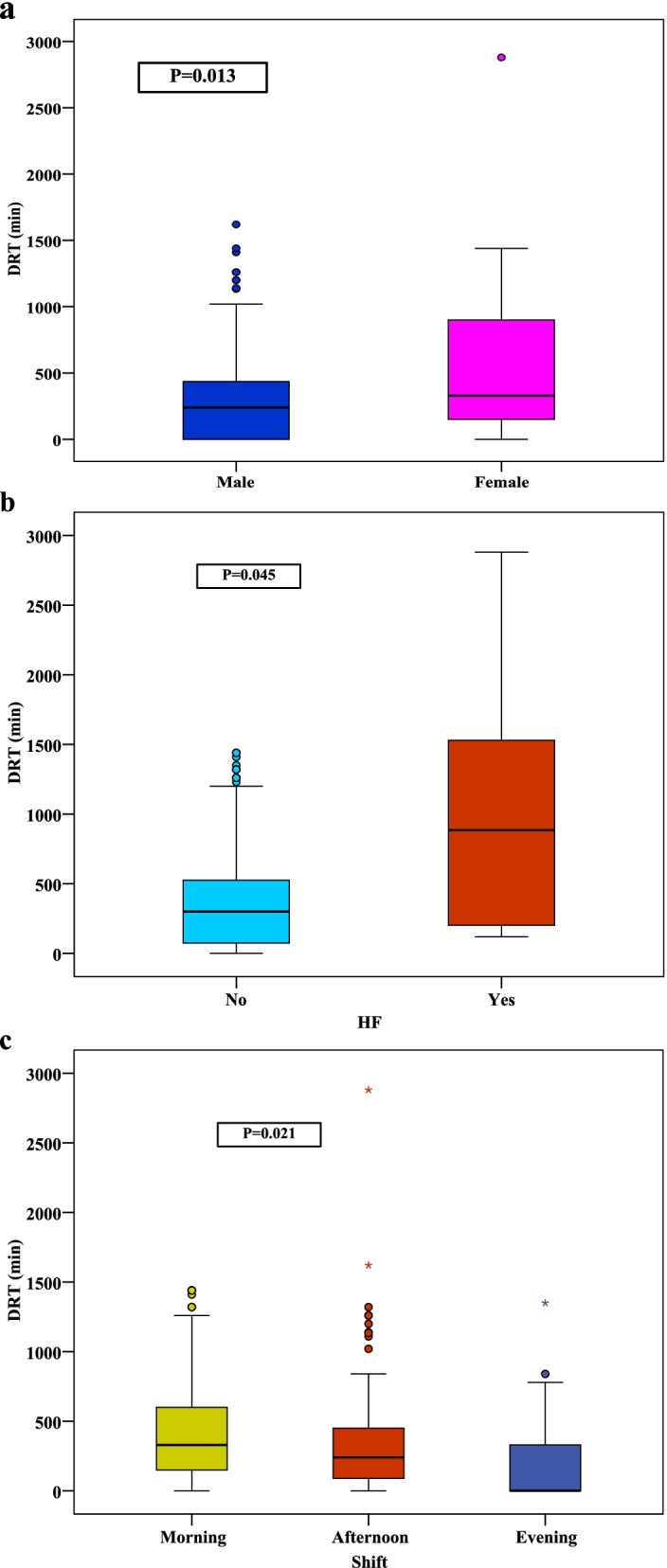
Associations between the DRT and sex, heart failure, and hemodialysis schedule. **a** Relation between DRT with sex. **b** Relation between DRT with heart failure. **c** Relation between DRT with hemodialysis schedule

There was a statistically significant positive correlation between the DRT and dialysate flow (*p* = 0.001), the number of missed sessions (*p* < 0.001), and the MIS (*p* = 0.001). On the other hand, there was a statistically significant negative relation between the DRT and age (*p* = 0.013), dialyzer surface area (*p* = 0.024), ultrafiltration (UF) volume (*p* = 0.012), UF rate (*p* = 0.013), dialysate Na (*p* = 0.006), post-HD MAP (*p* = 0.007), change in the MAP (*p* = 0.031), serum phosphate (*p* = 0.014), serum albumin (*p* = 0.048), symptom/ problem list (*p* < 0.001), EKD (*p* < 0.001), burden of kidney disease (*p* < 0.001), SF-12 physical composite (*p* = 0.001), and SF-12 mental composite (*p* < 0.001), Table 3. Males, heart failure patients, and patients of the morning HD shift had significantly prolonged DRT compared to females, patients with other comorbidities, and patients of the afternoon and evening HD shifts, respectively (Fig. 1).

**Table 3:** Association between the DRT and variable factors

DRT (min) vs.	r	p
**Age** (years)	− 0.179	0.013
**HD Vintage** (years)	0.104	0.153
**Frequency of HD** (per week)	− 0.082	0.261
**Missed sessions** (per month)	0.266	< 0.001
**Dialyzer surface area** (m^2^)	−0.164	0.024
**Blood flow rate** (ml/min)	0.031	0.666
**Duration per session** (hr.)	−0.026	0.721
**Ultrafiltration volume** (ml)	−0.181	0.012
**Ultrafiltration rate** (ml/hr.)	−0.180	0.013
**Dialysate temperature** (°c)	−0.044	0.547
**Dialysate Na** (mmol)	−0.198	0.006
**Dialysate flow** (ml/hr)	0.230	0.001
**KT/V Daugridas**	0.123	0.091
**URR**	0.130	0.072
**MAP** (mmHg)		
**Pre**	−0.047	0.516
**Post**	−0.194	0.007
**Change**	−0.156	0.031
**Hemoglobin** (g/dl)	0.016	0.822
**K** (mEq/L)	−0.083	0.255
**Na** (mEq/L)	−0.036	0.624
**Serum creatinine** (mg/dl)	0.014	0.847
**TIBC** (mcg/dl)	0.013	0.863
**Ca** (mg/dl)	0.034	0.637
**Ph** (mg/dl)	−0.178	0.014
**CRP** (mg/l)	−0.014	0.846
**Albumin** (g/dl)	−0.143	0.048
**MIS**	0.240	0.001
**Symptom/ problem list**	−0.392	< 0.001
**Effects of kidney disease**	−0.307	< 0.001
**Burden of kidney disease**	−0.270	< 0.001
**SF-12 physical composite**	−0.232	0.001
**SF-12 mental composite**	−0.353	< 0.001

*rs* Spearman coefficient

### Univariate and multivariate linear regression analysis

Regarding the DRT, a decrease in the dialyzer surface area, dialysate Na, and serum albumin were predictors of an increase in the DRT. Whereas, an increase in the dialysate flow, number of missed sessions, and MIS were predictors of an increase in the DRT.

All variables with *P* < 0.05 in the univariate analysis were included in the multivariate analysis. Dialysate Na, and number of missed sessions were independently correlated with the DRT (Table 4).

**Table 4:** Univariate and multivariate linear regression analyses for parameters affecting the DRT

	Univariate		^a^Multivariate	
	B (95%C.I)	p	B (95%C.I)	p
**Missed sessions** (per month)	95.036 (47.907–142.166)	< 0.001	91.180 (46.059–136.302)	< 0.001
**Dialyzer surface area** (m^2^)	− 378.278 (− 739.94 – −16.617)	0.040	− 168.505 (− 534.611–197.601)	0.365
**Dialysate Na** (mmol)	−38.961 (−63.284 – −14.637)	0.002	−34.993 (−58.277 – −11.709)	0.003
**Dialysate flow** (ml/hr)	0.598 (0.087–1.109)	0.022	0.485 (− 0.042–1.012)	0.071
**Albumin** (g/dl)	−177.121 (− 354.083 – − 0.160)	0.050	−88.745 (− 282.174–104.685)	0.367
**MIS**	21.696 (3.513–39.880)	0.020	17.817 (−2.075–37.709)	0.079

B: Unstandardized Coefficients

*C.I* Confidence interval *LL* Lower limit, *UL* Upper Limit, *MIS* Malnutrition inflammation score

^a^Multivariate analysis included the DRT as the dependent variable, while the number of missed sessions, dialyzer surface area, dialysate Na, dialysate flow, albumin, and MIS which showed significant association with DRT in univariate analysis were used as the independent variables

## Discussion

In our study, we identified factors affecting the DRT in our cohort and found that prolonged DRT was clearly associated with poorer HRQOL. Although post-HD fatigue commonly exists in dialysis patients, it is usually underestimated by physicians. Thus, appropriate and early identification of symptoms and associated factors might improve patients’ QOL. Extending research in this area will certainly be of great value to the HD population.

We found that dialysate Na and the number of missed sessions were independently correlated with the DRT as illustrated in the multivariate regression analysis. In this study, we found an inverse relationship between dialysate Na and the DRT. This is consistent with the findings of Rayner et al. [15] who reported that lowering the Na concentration in the dialysate (to 140 mEq/L) was associated with a longer DRT. Our findings suggest that patients’ symptoms during recovery may be partly due to disequilibrium. On the other hand, in the studies of Hussein et al. [27] and Bossola et al. [6], the dialysate sodium concentration did not differ significantly across the different recovery time categories. Also, the studies comparing high vs low dialysate sodium have revealed conflicting results [28, 29].

We demonstrated a positive correlation between the DRT and the number of missed sessions. This may be accounted for by the insufficient elimination of uremic toxins as a result of skipped HD sessions, which may contribute to the prolonged DRT in such individuals. Nevertheless, negative correlations were reported for KDQOL subscales and the number of missed sessions by Oliveira et al. [30] while missed treatments were associated with hospitalization, all-cause mortality, increased kidney disease burden, and poor mental and general health, according to Al Salmi et al. [31].

We demonstrated that malnutrition was associated with a prolonged DRT. We found a significant negative correlation between serum albumin level and the DRT (rs = − 0.143, *p* = 0.048), which is similar to the findings of Smokovska et al. [32] who reported that a shorter DRT was associated with a higher serum albumin level. On the other hand, Bossola et al. [6] found no significant correlation between serum albumin level and the DRT; however, they explained this by stating that all cases were managed till target albumin levels according to the KDOQI guidelines. Also, in our study, a significant positive correlation between the MIS and the DRT (rs = 0.240, *p* = 0.001) was found. We found no significant correlation between the DRT and the BMI (rs = − 0.129, *p* = 0.075), which is in line with the findings of Kodama et al. [5]. However, other researchers [15] found a longer DRT to be associated with a higher BMI. Our findings are expected because malnourished patients have increased rates of infections, hospitalization, morbidity and mortality. Also, we found a statistically significant positive correlation between the dialysate flow rate and the DRT. To the best of our knowledge, no research has looked into this association. But we have no clear explanation why these parameters (MIS, albumin and dialysate flow rate) failed to show an independent relationship with the DRT.

We found a negative significant correlation between the DRT and age (rs = − 0.179, *p* = 0.013), which is in line with the findings of Fitzpatrick et al. [33] and Yoowannakul et al. [34]. Other studies revealed no association between these variables [14, 32, 35, 36]. It is possible that the shorter recovery durations recorded by elderly individuals are associated with patients’ greater satisfaction and lower expectations despite having poorer clinical profiles. The average recovery time for male patients in this study was shown to be significantly shorter than that of female patients, which is in line with the findings of Rayner et al. [15]. According to recent research, females are more likely to develop unpleasant symptoms such as fatigue, exhaustion, and post-HD energy exacerbations than males [37, 38].

In the present work, we found no statistically significant correlation between the DRT and HD vintage. Similar findings were reported by other researchers [35, 39, 40]. However, these findings contradicted those of Rayner et al. [15], which revealed that dialysis vintage was associated with a longer recovery period. Also, we found that a patient’s hemodialysis schedule was associated with the DRT, where the evening shift had the lowest average, followed by the afternoon shift, and then the morning shift with the highest mean DRT. A possible explanation for this phenomenon is that patients in the evening shift go directly to sleep after returning home from the dialysis unit and wake up feeling better but omitting the sleep duration from the DRT.

We found no association between the DRT and the blood flow rate, which is in line with the findings of the RCT by Duggal et al. [41] which reported that a reduction in the blood flow rate did not ameliorate the DRT more than the usual care did. In the current study, we found no correlation between the DRT and the duration of each dialysis session, which is in line with the findings of Awuah et al. [35]. This may be because 165 out of 191 patients underwent four-hour long sessions, which may conceal any significant correlation. On the other hand, other researchers [15] found that patients reported longer DRTs with longer dialysis sessions.

We demonstrated that the DRT is inversely associated with the UF volume, UFR, and dialyzer surface area. Evidence of the association between the DRT and UFR is scarce and contradictory [6, 42, 43]. Bossola et al. [6] reported that the DRT and UFR are inversely related. Rayner et al. [15] reported that higher intradialytic weight loss was associated with a longer DRT. Furthermore, a U-shaped association between the recovery time and the UFR was identified, with both slow and rapid UFRs (5 and > 15 mL/min, respectively) being associated with a shorter DRT when compared to a UFR of 5–15 mL/min. On the other hand, Hussein et al. [27] reported that a greater UFR was associated with a longer DRT. Finally, Harford et al. [43] found no correlation between DRT and UFR. Bossola et al. [44] attributed this to significantly higher levels of interleukin-6 in HD patients with fatigue and hypothesized that the UFR may interfere with cytokine production or elimination.

We also discovered that the dialysate temperature had no effect on the recovery time. Similar findings were reported by Bossola et al. [6]. In the current work, we found no association between the pre-HD MAP and the DRT; however, we found a negative correlation between the post-HD MAP and the change in the MAP on one hand and the DRT on the other. Similar findings were reported by Yoowannakul et al. [45]. The drop in the blood pressure during HD leads to a reduction in the blood supply to the vital organs like the heart, brain, and mesenteries, which might explain the increase in the number of reports of backache with HD, in addition to the increased frequency of headache, dizziness, breathlessness, and cramps.

Also, we found no association between the DRT and the hemoglobin level, perhaps because our study population had a mean hemoglobin level of 9.8, which is close to the target hemoglobin (> 10) [46]. Many other studies reported similar results [47, 48]. However, Smokovska et al. [32] found a strong negative association between the Hb level and the post-HD recovery time. In our study, no correlation was found between serum calcium and the DRT, which is in line with the findings of McCann et al. [49] and Bossola et al. [6], who reported that patients’ phosphorus levels were not associated with fatigue. We found a significant negative correlation between serum phosphorus levels and the DRT, which suggests that low phosphorus levels may be associated with a patient’s malnutrition status.

The DRT was inversely associated with all five KDQOL subscales. Similar findings were reported by Rayner et al. [15] who stated that the DRT was inversely correlated with KDQOL measures.

The present study has some limitations. First, the younger age of the participants which may not represent the global dialysis population. Second, the DRT was assessed at a single point of time, not a mean of repeated measures. Third, this research was done in two dialysis centers in Alexandria University Hospitals and it would be better to include more centers. Fourth, a relatively small population was included (*n* = 191).

## Conclusion

Decreased dialysate Na, and increased number of missed sessions were independent predictors of prolonged DRT. Also, patients with prolonged DRT were associated with a poor quality of life. We recommend that DRT should be incorporated into the routine clinical evaluation of HD patients and, perhaps, utilized as an assessment measure of the dialysis treatment quality. We recommend that further studies, such as randomized clinical trials, should be conducted to examine strategies to minimize the DRT and, perhaps, enhance clinical outcomes, such as raising dialysate sodium concentration or decreasing dialysate flow.

## Data Availability

All data analyzed during this study are included in this manuscript.
